# News media framing of food poverty and insecurity in high-income countries: a rapid review

**DOI:** 10.1093/heapro/daad188

**Published:** 2023-12-27

**Authors:** Claire Kerins, Sinéad Furey, Páraic Kerrigan, Aodheen McCartan, Colette Kelly, Elena Vaughan

**Affiliations:** Health Promotion Research Centre, School of Health Sciences, University of Galway, University Road, Galway H91 TK33, Ireland; Department of Hospitality and Tourism Management, Ulster University Business School, Ulster University, Cromore Road, Coleraine, Co. Londonderry BT52 1SA, United Kingdom; School of Information and Communication Studies, University College Dublin, Belfield, Dublin 4 D04 V1W8,Ireland; School of Communication and Media, Ulster University, York Street, Belfast, Co. Antrim BT15 1ED, United Kingdom; Health Promotion Research Centre, School of Health Sciences, University of Galway, University Road, Galway H91 TK33, Ireland; Health Promotion Research Centre, School of Health Sciences, University of Galway, University Road, Galway H91 TK33, Ireland

**Keywords:** food poverty, food insecurity, food charity, framing theory, news media, rapid review

## Abstract

Food poverty and insecurity have become a public health emergency in many high-income countries. News media coverage can shape public and political views towards such issues. This rapid review synthesizes the evidence on how food poverty and insecurity are represented in the news media. Peer-reviewed publications were accessed through three electronic databases, with reference lists of all included studies screened. Primary research studies conducted in high-income countries and published in English since 1995 were included, with no restrictions on study methods. A combination of deductive coding to Entman’s framing theory and inductive analysis was used. Ten studies, mostly rated as low quality, were included in the review. Newspapers were the only type of news media examined. The findings showed a largely absent nuanced understanding of food poverty and insecurity, with the problem often defined by food bank use and the consequences mainly focused on physical health. The causes were mostly attributed to structural factors, with the solutions largely focused on charitable food aid. The discourse of recipient (un)deservingness of food aid was evident. Articles often contained views from government officials and charities, with individuals’ experiences of food poverty and insecurity largely absent. The findings of this review highlight that a major shift in print media discourse on food poverty and insecurity is required. More balanced and critical news reporting is required to present a more realistic picture of food poverty and insecurity, including its multi-dimensional nature, limitations of food charity and the need for structural solutions to this important issue.

**Contribution to Health Promotion**

- Food poverty and insecurity are recognized social determinants of health and are associated with significant adverse health outcomes.
- Given news coverage can shape public and political views, there is a need to understand how food poverty and insecurity are communicated in the news media.
- This evidence synthesis presents findings on how the news media define the problem, its respective causes, proposed solutions and moral judgements offered.
- The findings highlight a need for more critical news reporting on current approaches to combat food poverty and insecurity, and to acknowledge food as a human right and the need for government accountability.

## INTRODUCTION

Food poverty and insecurity are complex phenomena, which encompass multiple dimensions. While both terms refer to the same health and social issue, and are often used interchangeably in the literature, there are some differences in their definitions and associated dimensions. The term ‘food poverty’ is relatively new and can be defined as ‘the insufficient economic access to an adequate quantity and quality of food to maintain a nutritionally satisfactory and socially acceptable diet’ (O’Connor *et al*., 2016). It is comprised of four dimensions: economic access, quantity and quality of food, duration and social dimension (O’Connor *et al*., 2016). The term ‘food insecurity’ has been in existence for longer and can be defined as ‘the inability to consume an adequate quality or sufficient quantity of food in socially acceptable ways, or the uncertainty that one will be able to do so’ (Dowler *et al*., 2001). The Food and Agriculture Organization of the United Nations identified six dimensions of food security within their definition: food availability, food access (physical and economic), utilization, stability, agency and sustainability (HLPE, 2020). Despite differences in definition and dimensions, food poverty and food insecurity are considered interrelated concepts (O’Connor *et al*., 2016).

Food poverty and insecurity remain relatively hidden, especially in high-income countries where there is a perception that it is not a problem (Drew, 2022; BMC Medicine Editorial, 2023). Food poverty exists in the Global North and the right to food continues to be compromised by poverty due to unexpected changes in employment and household bills combined with a lack of savings for emergency expenditures (Nord and Brent, 2002; Nord, 2009; Olabiyi and McIntyre, 2014; Kleve *et al*., 2018). Recent studies have highlighted how food poverty is a rising problem in affluent societies, ranging from 8% to 20% of the population (Pollard and Booth, 2019), whereby people cannot afford to eat a nutrient-dense, healthy diet for reasons of unaffordability—as measured by food deprivation measures (Davis and Geiger, 2017), thereby emphasizing poverty as a key driver of food insecurity.

In the European Union (EU), one of the wealthiest regions on Earth, 22% of its population—95.3 million people—are classified as at risk of poverty (Eurostat, 2023). Data from the EU show that 19.7% of those at risk of poverty were unable to afford a meal containing meat, fish, or a vegetarian equivalent in 2022, an increase of 12.6% on 2021 (Eurostat, 2023). This increase is likely reflective of the current cost-of-living crisis triggered by the aftershock of the COVID-19 pandemic (Devereux *et al*., 2020) and exacerbated by the inflationary impact of the Russia–Ukraine conflict (Maurya *et al*., 2023). The growth of this problem represents a reversal of progress towards meeting the United Nations Sustainable Development Goal (SDG) 1 (No poverty), with inevitable knock-on consequences for SDG 3 (Good health and well-being) (Lafortune *et al*., 2022). Absent of intervention however, food poverty and insecurity will likely continue to affect the health and well-being of a significantly greater number of individuals and families, particularly given the on-going threat of climate change to global food security (Porter *et al*., 2014).

The health consequences of food poverty and insecurity have, by now, been well documented. Indeed, given the intersection of food poverty and insecurity with a cluster of chronic and disparate, yet inter-connected, health conditions, public health scholars have argued that food poverty and insecurity, and ensuing malnutrition, constitutes a syndemic in which social and health inequalities mutually reinforce one another (Swinburn *et al*., 2019; Himmelgreen *et al*., 2022). A significant body of evidence would appear to support this:

Several meta-analyses have shown that food insecurity in adults is associated with micro-nutrient deficiency and increased risk of anaemia (Lopes *et al*., 2023); obesity and myocardial infarction (Salinas-Roca *et al*., 2022); both inadequate and excessive weight gain in pregnancy (Arzhang *et al*., 2022); and overall multi-morbidity (Kantilafti *et al*., 2023). Among older adults, food poverty is associated with non-adherence to medication, poor mental health outcomes and poor physical functioning (Assoumou *et al.*, 2023), while other studies suggest links to increased risk of diabetes, hypertension and higher odds of chronic disease (Pooler *et al*., 2019).

Children and adolescents experiencing food poverty are less likely to report eating fruits, vegetables and healthy grains and more likely to report eating unhealthy foods (Molcho *et al*., 2007), including increased consumption of fast-food (Smith *et al*., 2022). Evidence from one longitudinal study suggests that early exposure to food insecurity is associated with a higher BMI in children from 2 years of age, putting them at increased risk of over-weight and obesity in later life (Zhong *et al*., 2022). Food poverty among children is further associated with cognitive problems, anaemia, aggression and anxiety, higher risk of hospitalization, and poorer mental, oral and general health (Gundersen and Ziliak, 2015).

There is evidence also to suggest that food poverty may have negative mental health consequences. A meta-analysis of the association of food insecurity with mental health outcomes in parents and children, which pooled data from studies (*n* = 108) of 250,553 parents and 203 822 children, reported significant associations between food poverty and parental depression, anxiety and stress, and between food insecurity and child depression, externalizing/internalizing behaviours and hyperactivity (Cain *et al*., 2022). Furthermore, analysis of pooled data from 67 countries in low-, middle- and high-income countries world-wide has shown that adolescent girls (*n* = 121,248) aged 11–17 who have ever experienced food insecurity had significantly higher odds of reporting suicidal behaviour (Mahumud *et al*., 2022).

### Health, policy and the media

With food poverty and insecurity apparently on the increase in Europe as a result of the convergence of multiple, intersecting global crises, there is (and will continue to be) a concurrent increase in media reporting on the issue. Media reporting is a primary source of information for the public on social- and health-related issues such as food poverty and insecurity (Picard and Yeo, 2011). While public opinion is rarely homogenous on any issue, and with the media frequently reflecting public viewpoints through polls, vox pops and coverage of mass and popular movements (Beckers and Moy, 2023), both print and broadcast media still play an active role in shaping public opinions (Happer and Philo, 2013). The news media have an acknowledged and key role in agenda setting (McCombs, 2005), as gatekeepers of macro-level discourse with the power and influence to shape and constrain policy debates. Editorial and journalistic choices about which aspects of an issue to highlight and make salient serve to define problems and suggest and legitimise specific solutions or approaches (Entman, 1993), often along dominant ideological lines (Vaughan and Power, 2023). How food poverty and insecurity are framed in the media is consequently of importance in understanding how both public and policy-makers’ knowledge about the issue is constituted.

Furthermore, identifying how an issue such as food poverty and insecurity is framed can give insight into beliefs, suppositions or assumptions about the issue, in respect of its causality, its response and who is responsible for addressing it. Framing, as articulated and defined by Entman, involves a process of simplification of an issue in which the communicator selects aspects of an issue to make salient or ‘more noticeable, meaningful or memorable to audiences’ (1993). With many pressing global health problems, insights on which aspects of complex problems are made salient is useful for understanding how some issues can attract greater attention, funding, collective action and policy prioritization, while other comparable issues lack the same urgency and commitment (Shiffman and Shawar, 2022). Given the well documented health impacts of food poverty and insecurity, having a better understanding of how the issue of food poverty and insecurity is currently framed can help health promoters, researchers and other stakeholders to analyse and strategize new ways to communicate and advocate for more meaningful responses.

Several studies have explored how food poverty and insecurity is communicated in the mass media, however to date there have been no evidence syntheses of such studies. This rapid review aims to address this gap by systematically gathering, synthesizing and critiquing the extant literature on news media representations of food poverty and insecurity in high-income countries. Such a synthesis and analysis would be useful in helping to better understand how the issue of food poverty and insecurity, and responses to it, have thus far been constructed in the media, and may shed light on alternate framings which could help inform the work of health promoters and communicators to advocate for effective policy measures.

## METHODS

A rapid review was conducted to provide a summary of the literature in a timely and resource-efficient manner (Moons *et al*., 2021). Such reviews streamline components of the systematic review process to enable a shorter lead-time to help inform programmes of research, as well as provide guidance for practitioners and policy-makers (Munn *et al*., 2015). In this case, the rapid review was carried out as part of a wider project exploring communication of food poverty on the island of Ireland, the overall aim of which is to better understand how food poverty is discursively constructed and how public, policy and journalistic stakeholders perceive the topic. While no reporting guideline for rapid reviews has been developed yet, the Preferred Reporting Items for Systematic Reviews and Meta-Analyses (PRISMA) guidelines (Page *et al*., 2021) were followed in drafting this review (see Supplementary Additional File 1). A protocol was prepared in advance and published on the Open Science Framework website (Kerins *et al.*, 2023).

### Eligibility and search criteria

The criteria for study eligibility are summarized in Table 1. As per the protocol (Kerins *et al.*, 2023), we initially planned to include studies of news media framing of food poverty; however, we decided to expand the outcome of interest to include food insecurity. This decision was based on food poverty and insecurity being interrelated concepts and both terms often used interchangeably in the literature (O’Connor *et al*., 2016). All primary research studies conducted in high-income countries (as per the Organisation for Economic Co-operation and Development, OECD, definition (The World Bank, 2019) using qualitative, quantitative, mixed-method and multi-method approaches were eligible for inclusion. Peer-reviewed journal articles published in English since 1995 were included in the review. This timeframe was chosen based on when food insecurity was first assessed by the most prolific/dominant measure (USDA HFSSM Measure (Rafiei *et al*., 2009)).

**Table 1:** Study eligibility criteria

	Inclusion criteria	Exclusion criteria
Population	Individuals or households living with or at risk of food poverty and insecurity. No restrictions on target population or age group. Other terms used to describe food poverty and insecurity are included, for example, food deprivation, food insufficiency and hunger.	
Outcome	News media framing of food poverty and insecurity. The term news media refers to any media that provides news or information to the public, including print media (newspapers, magazines), broadcast news (TV and radio news) and online news sites (digital newspapers).	Social media Findings from reader-generated online comments in response to news articles and other readers.
Study type	All primary research studies (from peer-reviewed literature) using qualitative, quantitative, multi-methods or mixed methods approaches.	Grey literature Editorials, commentary and opinion pieces
Publication year	1995 onwards	Before 1995
Setting	High-income countries	Non-high-income countries
Language	English	Languages other than English

The search was conducted in Medline, Scopus and PsycINFO electronic databases using keywords, synonyms and subject headings (where available) in February 2023. A university librarian reviewed and provided feedback on the search strategy to optimize sensitivity and specificity. The search strategy is presented in Supplementary Additional File 2. Screening reference lists of all included studies were undertaken.

### Study selection and appraisal

Search results were exported into EndNote, where duplicates were removed. Following this, search results were imported into Rayaan, an online screening platform, for title and abstract screening. Two reviewers independently screened 23% of titles and abstracts, with one reviewer screening the remaining titles and abstracts. Following this, two reviewers dual screened 20% of full-text articles in Microsoft Excel, with the remaining full-text articles screened by one reviewer. A second reviewer screened all excluded papers. Any disagreements were discussed and resolved.

The methodological quality of all included studies was assessed using the Mixed Methods Appraisal Tool (MMAT) version 2018 for quantitative, qualitative and mixed methods research designs (Hong *et al*., 2018). For each included study, a methodological rating (i.e. 0, 20, 40, 60, 80 or 100) was calculated using the MMAT. Two independent reviewers rated 30% of the included studies, with disagreements resolved through discussion. One reviewer assessed the quality of the remaining studies. No estimates of inter-rater reliability for screening and quality assessment were calculated.

### Data extraction and synthesis

One reviewer extracted data from each study using a data extraction form in Microsoft Excel. Extracted data included key study information (name of the first author, year of publication, country of study, study focus, study methods, source of news media data, type of news media, data collection period, analysis methods) and findings on news media framing of food poverty and insecurity. Data on news media framing of food poverty and insecurity were extracted from the results and discussion sections of the included studies. The rationale for extracting data from the results and discussion sections is based on findings that raw data from qualitative research may be presented in both sections (Booth *et al*., 2016; Boland *et al*., 2017).

Data synthesis was led by one reviewer using a mix of inductive and deductive analysis techniques, with consultation with review team members through a consensus decision-making process (Hill *et al*., 1997, 2005). As part of the deductive coding process, findings on news media framing of food poverty and insecurity were mapped to Entman’s framing theory. To frame, according to Entman, is ‘to select some aspects of a perceived reality and make them salient in communicating text’ (1993). Entman’s conceptualization of framing consists of the following elements: defining the problem, identifying the respective causes, proposing solutions to address them and offering a moral evaluation (Entman, 1993). This offers a useful framework for breaking down and analysing the constitutive parts of the discursive construction of complex issues. It is also a more accessible communication framework than approaches such as, for instance, Discourse Analysis, which have denser theoretical underpinning and relies on specialized knowledge of socio-linguistics and pragmatics. To capture themes within Entman’s framing functions, inductive thematic analysis was performed (Saldana, 2021). The review findings were synthesized narratively and using tabulation.

## RESULTS

**Fig. 1:**
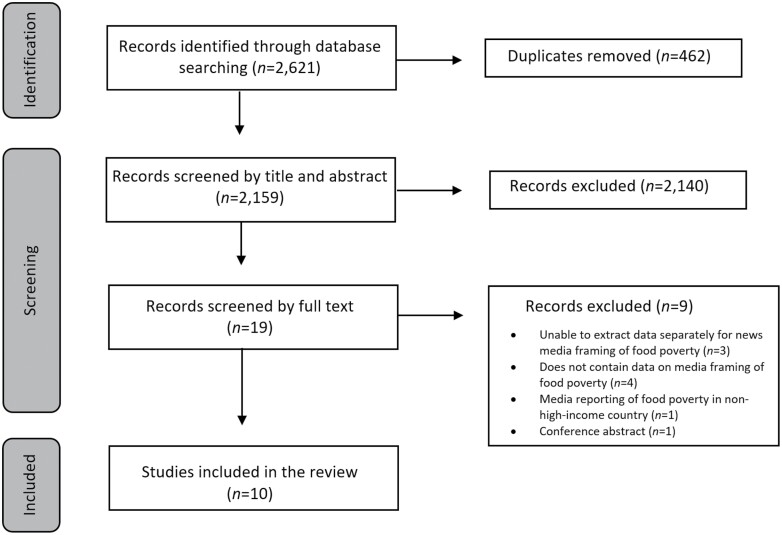
PRISMA flow diagram of study selection process.

Our search identified 2159 potentially eligible studies after duplicates were removed. Of these, 10 were included in the review (Figure 1). Table 2 provides a description of study characteristics. Studies used mostly qualitative data collection methods (*n* = 9), followed by quantitative (*n* = 1). Newspapers were the only news media type examined within our sample, with most studies (*n* = 9) focused on national newspapers. Print newspapers were examined across half of the studies (*n* = 5), with online newspapers used in two studies and the newspaper format not specified in the remaining studies (*n* = 3). The timeframe in which news articles were retrieved ranged between 2006 and 2020. The majority of studies (*n* = 5) examined newspaper coverage of food assistance programmes (e.g. food banks), while the remaining studies examined newspaper coverage of food insecurity/poverty (*n* = 4) and rising food costs (*n* = 1). Four of the included studies were from the UK. The remainder were from Canada (*n* = 2), the US (*n* = 1), Australia (*n* = 1), Spain (*n* = 1) and Finland (*n* = 1). In terms of study quality, the majority of studies (*n* = 7) were rated as low quality, with an MMAT score of 60 or lower. Most studies were rated as low quality due to inadequate reporting to allow for assessment against specific methodological quality criteria. Detailed study quality analysis is presented in Supplementary Additional File 3.

**Table 2:** Characteristics of included studies (*n* = 10)

First author, publication year	Country of study	Study focus	Study methods	Data source	Type of news media (*n*)	Data collection period	Analysis	MMAT score,
Collins *et al*., 2016	Canada	Newspaper coverage of household food insecurity in Canada	Quantitative descriptive	News articles via online media database	National (*n* = 2) and local/regional (*n* = 16) newspapers (print)	2007–12	Content analysis	60
Henderson and Foley, 2010	Australia	Newspaper coverage of rising food costs in Australia	Qualitative	News articles via online media database	National newspapers (print) (*n* = 5)	2007–08	Content analysis	40
Knight *et al*., 2018	UK	Newspaper coverage of food poverty as experienced by UK children and families	Qualitative^a^	News articles via online media database	National newspapers (print) (*n* = 6)	2006–15	Thematic and narrative analysis	80
Marín-Murillo *et al*., 2020	Spain	Newspaper coverage of hunger/food accessibility in Spain	Qualitative^a^	News articles via online media databases	National newspapers (online) (*n* = 4)	2017	Content analysis and the framing theory	40
Mejia *et al*., 2022	US	Newspaper coverage of food assistance before and during the COVID-19 pandemic in the US	Qualitative	News articles via online media databases, and photographs associated with articles via Google search engine	National newspapers (*n* not reported)	2019–20	Ethnographic content analysis	20
Price *et al*., 2020	UK	Newspaper coverage of foodbank use in the West Midlands, UK	Qualitative	News articles via online media database	Local newspapers (online) (*n* = 3)	2010–19	Grounded theory approach	40
Smith-Carrier, 2021	Canada	Newspaper coverage of Christmas food hamper program in Canada	Qualitative	News articles via news media websites and Google search engine	National newspapers (*n* not reported)	2009–19	Discourse-historical approach of critical discourse analysis	80
Tikka, 2019	Finland	Newspaper coverage of charitable food aid in Finland	Qualitative	News articles via newspaper digital archive	National newspaper (*n* = 1)	1995–2016	Frame package analysis	20
Wells and Caraher, 2014	UK	Newspaper coverage of the foodbank phenomenon in the UK	Qualitative^a^	News articles via online media database	National newspapers (print) (*n* = 9)	1993–2014	Thematic analysis	40
Yau *et al*., 2021	UK	Newspaper coverage of food insecurity in UK	Qualitative^a^	News articles via online media database	National newspapers (print and online) (*n* = 12)	2016–19	Thematic analysis	100

^a^Qualitative data extracted from a multi-methods study.

### News media framing of food poverty and insecurity

A summary of key findings on news media framing of food poverty and insecurity mapped to Entman’s theory for each individual study is presented in a table in Supplementary Additional File 4. A narrative summary of the findings on news media framing of food poverty and insecurity is presented below.

#### Definitions of the problem of food poverty and insecurity

Eight out of 10 studies reported findings on how the problem of food poverty and insecurity was defined and/or the consequences of food poverty and insecurity in newspaper coverage. Across three of these studies, food bank usage was synonymous with food poverty and insecurity, and linked with poor dietary quality (Knight *et al*., 2018; Marín-Murillo *et al*., 2020; Yau *et al*., 2021). One of these studies found that insecurity was largely defined as inadequate food quantity and that the term ‘holiday hunger’ was commonly used to define the extent and timing of child food poverty (Yau *et al*., 2021). One study found few articles (8%) connecting food access and health (Mejia *et al*., 2022). Four studies reported newspaper coverage of physical health implications such as obesity and malnutrition (Henderson and Foley, 2010; Knight *et al*., 2018; Marín-Murillo *et al*., 2020; Yau *et al*., 2021). Fewer studies (*n* = 2) reported coverage of mental health consequences (Price *et al*., 2020; Yau *et al*., 2021). Only one study found coverage of social health consequences, and the educational consequences for children living with food insecurity (Yau *et al*., 2021). One study reported on newspaper coverage of the mortality risk of food poverty such as a shorter life expectancy (Marín-Murillo *et al*., 2020).

#### Causal attributions of food poverty and insecurity

Nine out of 10 studies reported findings on the drivers of food poverty and insecurity presented in newspaper coverage. Across most studies, the immediate drivers of food poverty and insecurity were reported as low income (Wells and Caraher, 2014; Knight *et al*., 2018; Yau *et al*., 2021; Mejia *et al*., 2022) and high cost-of-living (Henderson and Foley, 2010; Wells and Caraher, 2014; Knight *et al*., 2018; Yau *et al*., 2021), resulting in insufficient income (Wells and Caraher, 2014; Knight *et al*., 2018; Tikka, 2019; Price *et al*., 2020; Yau *et al*., 2021; Mejia *et al*., 2022). Newspaper coverage of upstream drivers of food poverty and insecurity, or charitable food aid use were largely governmental (Wells and Caraher, 2014; Knight *et al*., 2018; Tikka, 2019; Price *et al*., 2020; Yau *et al*., 2021). Governmental drivers included austerity measures taken during economic recession (Knight *et al*., 2018; Tikka, 2019; Yau *et al*., 2021) and issues with the welfare system (such as inadequate social security payments, sanctions, delays in payment) (Wells and Caraher, 2014; Knight *et al*., 2018; Price *et al*., 2020; Yau *et al*., 2021).

There was some reference to individual responsibility across studies, such as lack of money management and cooking skills (Wells and Caraher, 2014; Knight *et al*., 2018; Marín-Murillo *et al*., 2020; Smith-Carrier, 2021; Yau *et al*., 2021). However, in one study, individual responsibility was often mentioned in the context where politicians were criticized for viewing food insecurity as individual failing (Yau *et al*., 2021). Only two studies found newspaper coverage of food insecurity or food charity use framed as a problem of individual nature, with no or limited reference to structural causes (Marín-Murillo *et al*., 2020; Smith-Carrier, 2021).

In one study, the economic impact of the COVID-19 pandemic was reported as an upstream driver of increased use of food assistance (Mejia *et al*., 2022). This study also found few articles linking structural inequities to food insecurity. Across two studies, the increased supply of food banks was reported as a driver of food bank use in newspaper coverage (Wells and Caraher, 2014; Knight *et al*., 2018). One study found when charitable food aid was reframed as a circular economy model (to address food waste), the root causes for food insecurity were not addressed in newspaper coverage (Tikka, 2019).

#### Moral evaluations of food poverty and insecurity

All included studies considered how the problem of food poverty and insecurity were perceived or presented in newspaper coverage. Across most studies (*n* = 5), newspaper coverage of food poverty and insecurity, or food bank use was used to illustrate poverty (Wells and Caraher, 2014; Tikka, 2019; Marín-Murillo *et al*., 2020; Price *et al*., 2020; Yau *et al*., 2021). In two studies, the existence of food poverty and insecurity were presented as incompatible with modern day living, with reference to periods of poverty in British history (Wells and Caraher, 2014; Yau *et al*., 2021). Recipients of food assistance were presented as mainly families in need (Marín-Murillo *et al*., 2020; Smith-Carrier, 2021; Mejia *et al*., 2022), with one study finding more reference to families in need during the COVID-19 pandemic than pre-pandemic (Mejia *et al*., 2022). Other studies reported those at risk of food insecurity being presented as the ‘working poor’ (Yau *et al*., 2021) and the ‘average Australian’ being impacted by rising food prices (Henderson and Foley, 2010). Two studies reported food poverty being framed as a rights issue for children, although only referenced in a few newspaper articles (Knight *et al*., 2018; Marín-Murillo *et al*., 2020). One study reported the absence of any mention of human rights in reporting on food insecurity (Smith-Carrier, 2021).

The ‘(un)deserving’ poor narrative was evident across five studies (Wells and Caraher, 2014; Knight *et al*., 2018; Price *et al*., 2020; Smith-Carrier, 2021; Mejia *et al*., 2022). Recipients deemed more deserving of assistance included those seeking work (Knight *et al*., 2018; Mejia *et al*., 2022), in difficult circumstances (Knight *et al*., 2018; Price *et al*., 2020; Mejia *et al*., 2022), parents of young children (particularly lone parents) (Smith-Carrier, 2021; Mejia *et al*., 2022) and university students (considered future employees/workers) (Smith-Carrier, 2021). One study found food assistance recipients to be more deserving of support during the COVID-19 pandemic (with recipients cast as desperate and ‘suddenly hungry’—portrayed empathetically as ‘people like us’ in news coverage) than before the pandemic (with reference to fraudulent behaviour) (Mejia *et al*., 2022). Other studies also found charitable food aid described as vulnerable to misuse by those undeserving, casting doubt on the needs and motives of food bank users (e.g. opportunistically taking advantage, freeloaders abusing the service) (Wells and Caraher, 2014; Knight *et al*., 2018; Smith-Carrier, 2021). One study found no undeserving discourse, with empathy and compassion for food bank recipients evident in newspaper reporting (Price *et al*., 2020).

Government denial of the problem and their responsibility was reported across two studies (Knight *et al*., 2018; Yau *et al*., 2021). For example, one study found media coverage of the government accusing the Trussell Trust of ‘scaremongering’, with statistics on food poverty being questioned (Knight *et al*., 2018). The Trussell Trust is a UK-based NGO that supports a network of food banks while working and campaigning to end the need for food banks. Another study found a negative tone was adopted when senior governments were implicated in actions to address food insecurity, compared to more neutral and positive tones when local/municipal governments were profiled (Collins *et al*., 2016).

Across most studies (*n* = 7), newspaper reporting on charitable food aid was predominately positive (Wells and Caraher, 2014; Collins *et al*., 2016; Knight *et al*., 2018; Tikka, 2019; Price *et al*., 2020; Smith-Carrier, 2021; Yau *et al*., 2021). When food banks were framed as distributors of food waste it was considered a double win for both the environment and food poverty (Tikka, 2019; Yau *et al*., 2021). Findings from another study showed food banks presented as a potential ‘partner’ of the welfare state (Knight *et al*., 2018). Across two studies, food banks were presented as an example of government’s ‘Big Society’ [the ‘Big Society’ was a flagship policy of the UK Conservative Party in their manifesto for the 2010 general election; Big Society was posited by the Conservative Party as an initiative to increase volunteerism in communities but was widely criticized as a measure aimed at further shrinking essential public services in line with the policy of austerity specifically and neoliberalism more broadly (Kisby, 2010)] in action (Knight *et al*., 2018; Price *et al*., 2020). One study reported on the assumed adequacy and venerated practice of charitable food aid in newspaper coverage (Smith-Carrier, 2021). The challenges faced by charitable initiatives (e.g. getting enough volunteers, funding) and their success (e.g. awards) were presented across two studies (Marín-Murillo *et al*., 2020; Mejia *et al*., 2022).

Some studies (*n* = 4) reported negative coverage of charitable food aid (Knight *et al*., 2018; Marín-Murillo *et al*., 2020; Yau *et al*., 2021; Mejia *et al*., 2022). One such study found articles highlighting that charitable initiatives were unsustainable and addressed the symptoms rather than the root causes of food insecurity (Yau *et al*., 2021). Another study reported food banks being portrayed as the ‘enemy’ in creating welfare dependency (Knight *et al*., 2018). In another study, news coverage of difficulties in accessing charitable food aid (e.g. complex application process) were reported (Mejia *et al*., 2022). One study reported interest-driven collaborations in charitable food aid presented in news coverage, in which the private sector contributed to the cause at the same time as advertising themselves (Marín-Murillo *et al*., 2020).

Narratives surrounding ‘Big Society’ and citizens playing an active role in their community (i.e. donating to or volunteering at food banks) was reported (Knight *et al*., 2018; Price *et al*., 2020). One study reported the unequal power relations, with volunteers (givers/donors) being framed as the saviour or rescuer of ‘poor’ people (Smith-Carrier, 2021). The same study found the rewards for volunteers (e.g. feelings of joy, pleasure and fulfilment) evident in media reporting, while the emotional costs (e.g. experiences of anger, shame, humiliation and degradation) for recipients omitted (Smith-Carrier, 2021). Furthermore, this study reported volunteer expectations of gratitude and preferred behaviours (e.g. demonstrate the ‘Christmas spirit’ expected of them) from food charity recipients (Smith-Carrier, 2021).

#### Solutions to address food poverty and insecurity

All the included studies reported findings on existing or proposed solutions to food poverty and insecurity in newspaper coverage. Existing solutions to address food poverty and insecurity were predominantly solidarity initiatives (*n* = 8) (Wells and Caraher, 2014; Collins *et al*., 2016; Knight *et al*., 2018; Tikka, 2019; Marín-Murillo *et al*., 2020; Price *et al*., 2020; Smith-Carrier, 2021; Yau *et al*., 2021), with charity-based solutions (such as food banks and redistribution of food waste initiatives) reported in six of these studies (Wells and Caraher, 2014; Knight *et al*., 2018; Tikka, 2019; Price *et al*., 2020; Smith-Carrier, 2021; Yau *et al*., 2021). The Trussell Trust was presented as the model of operation for food banks in one study (Wells and Caraher, 2014). Fewer studies reported existing or proposed structural solutions to address food poverty and insecurity (Collins *et al*., 2016; Yau *et al*., 2021; Mejia *et al*., 2022). One such study found local/municipal governments (as opposed to senior governments) frequently implicated in actions to address food insecurity (Collins *et al*., 2016). Another study found the number of articles that proposed government policy solutions more than tripled during the COVID-19 pandemic (Mejia *et al*., 2022). Calls for government actions were reported in another study, with advocacy for structural income-based solutions (e.g. welfare reform, increased minimum wage) (Yau *et al*., 2021). There was some mention of solutions that were individual (skills-based e.g. budgeting skills and cost-saving strategies e.g. buying in bulk, packing lunches) across two studies (Henderson and Foley, 2010; Yau *et al*., 2021).

#### Social actors involved in the discourse

A large proportion of the included studies found articles often containing views from government officials/politicians (*n* = 6) (Henderson and Foley, 2010; Wells and Caraher, 2014; Knight *et al*., 2018; Tikka, 2019; Yau *et al*., 2021; Mejia *et al*., 2022) and charities (namely food banks) (*n* = 5) (Wells and Caraher, 2014; Knight *et al*., 2018; Smith-Carrier, 2021; Yau *et al*., 2021; Mejia *et al*., 2022). Other actors involved in the discourse included advocacy groups (*n* = 3) (Knight *et al*., 2018; Yau *et al*., 2021; Mejia *et al*., 2022), journalists/media (*n* = 3) (Henderson and Foley, 2010; Knight *et al*., 2018; Tikka, 2019), church leaders (*n* = 3) (Wells and Caraher, 2014; Knight *et al*., 2018; Tikka, 2019), other public figures (*n* = 2) (Wells and Caraher, 2014; Knight *et al*., 2018), private sector (*n* = 2) (Tikka, 2019; Marín-Murillo *et al*., 2020) and non-governmental organizations (*n* = 1) (Marín-Murillo *et al*., 2020). Only two studies reported good representation of individuals experiencing food insecurity or food bank users in news coverage (Price *et al*., 2020; Yau *et al*., 2021). Four studies found the voices of those affected by food poverty and insecurity to be largely absent (Wells and Caraher, 2014; Knight *et al*., 2018; Tikka, 2019; Marín-Murillo *et al*., 2020). Two studies found other groups (e.g. food bank volunteers) often acted as a proxy voice for food bank users (Wells and Caraher, 2014; Knight *et al*., 2018).

## DISCUSSION

### Summary of key findings

This is the first review to synthesize the evidence on news media framing of food poverty and insecurity in high-income countries. The review findings demonstrate that research on news media framing of food poverty and insecurity is limited to newspapers only, with studies largely focused on national print newspapers. Research conducted is further limited to a small number of high-income countries (6 out of the 38 OECD member countries), with the largest proportion of studies conducted in the UK. Reporting on the issue of food poverty and insecurity across all countries was remarkably homogenous; one study noted differences, however, between reporting prior to and after the COVID-19 pandemic. The results of the evidence synthesis highlight a heavy reliance on food bank usage as the proxy definition of food poverty and insecurity (i.e. relating to insufficient food quantity), with a lack of or limited reference to other dimensions (e.g. poor diet quality, reduced social participation). The physical health implications of food poverty and insecurity were evident in news media reporting, with limited coverage of the mental and social health consequences. The immediate and upstream drivers of food poverty and insecurity were mostly framed as insufficient income (due to low income and high cost-of-living) and governmental (due to austerity measures and issues with welfare system), respectively. There was some, but limited, reference to individual responsibility (e.g. lack of money management and cooking skills). Food poverty and insecurity were built through the media as a problem that impacts the poor, where the solutions are left to the charitable good will of volunteers and donors. The discourse of deservingness was evident, in which deservingness of food assistance was contingent on willingness to pursue paid work or membership of groups such as families (particularly lone parents) caring for young children. The reported existing solutions predominantly focused on solidarity initiatives such as charitable food aid, with less coverage of structural/policy solutions to address food poverty and insecurity. Articles often contained views from government officials and charities (namely, food bank organizers and volunteers), while the voices of those affected by food poverty and insecurity, health and academic experts were largely absent.

### Interpretation and implications

A nuanced understanding of food poverty and insecurity was not reflected in the reported solutions, which relied heavily on charity-based solutions. Food bank use was seen as synonymous with food poverty and insecurity in media discourse. A narrative surrounding civil society and the important role of volunteers and charities in helping those in need was evident across media coverage. This narrative is problematic as ‘charity in the form of food banks and food aid is highly depoliticizing’ (Caplan, 2016). This and other problems associated with food charity (such as food banks and redistribution of food waste initiatives) have been well documented in the literature (e.g. addressing symptoms, limited food choice, poor food quality, costs to human dignity, food waste solutions promoting unsustainable food production, etc.) (Poppendieck, 1999; Salonen, 2016; Kortetmäki and Silvasti, 2017; Smith-Carrier *et al*., 2017) but were not evident in news media reporting. One possible explanation for the absence of a critical lens regarding current approaches to combat food poverty and insecurity may be the economic pressures on the journalism model, which could be contributing to the failure of time-pressed journalists to properly interrogate such issues (Lewis *et al*., 2008). On the other hand, the limited focus on government accountability may reflect suggestions that news media is an important part of national and global elites and tends to conserve the interests of the more powerful groups in society (Seale 2003; Collins *et al*., 2006). Certainly, it was evident in the findings that the voices of politicians were prominent in news media coverage of food poverty and insecurity.

A key finding of the review is the inherent discordance, if not outright contradiction, in a media narrative that acknowledges upstream factors (i.e. austerity, welfare policy, macro-level economic conditions, etc.) as the key drivers of food poverty and insecurity, while focusing solely on down-stream measures as the solution. One possible explanation may be that poverty, more broadly, represents a complex ‘wicked problem’, to which the solution is neither straightforward nor politically or ideologically uncontested (Pradilla *et al*., 2022). This may be compounded by a newsroom culture in which readers are often perceived as desiring to read about immediate and concrete solutions rather than abstract, longer term and technically complex policy strategies. Bastian (2011), for instances, argues that readers like tangible answers to problems in order to give them a sense of control and power over their lives. This thinking arguably impacts on how the news media frames complex social issues and approaches to addressing them (Wallack, 1993). A further possible factor contributing to the contradictory narrative of down-stream solutions to upstream problems is the relative absence of the expert voices of academics, researchers and health professionals, whose input to the discourse may act to provide authoritative countervailing points of view.

Also absent to a significant extent were the voices of those living in food poverty and insecurity. This may be explained by unwillingness on the part of individuals living in food poverty and insecurity to be interviewed, gatekeeping of service-users by food aid organizers, or a reluctance on the part of journalists to approach vulnerable individuals. While in theory including the voices of vulnerable populations should be encouraged, in practice this may be somewhat ethically fraught. Wells and Caraher (2014) found that positive news coverage (e.g. hard work of volunteers, awards achieved) was provided at the expense of the voices of food bank users. When food bank users were quoted it was most often to explain their circumstances and/or express gratitude for the food they have received (Wells and Caraher, 2014). Chauhan and Foster (2014) have pointed out that people living in poverty are frequently othered in the media through representational absence and denial. Such othering, they argue, acts as a rhetorical salve to middle-class sensibilities that ‘restores the notion of general prosperity and well-being in society while the significance of the problem, and any attempt to deal with it, dwindle’ (Chauhan and Foster, 2014). Indeed, consideration should be given to the relative merits and demerits of the perceived necessity of including experts by experience in all food insecurity portrayals. There may be occasions when the lived experience helpfully demonstrates exactly the point to be made while at other times it may represent a disservice to the bigger picture where the prevalence statistics should suffice to detail the social policy problem without demoting the issue to an individual experience.

Largely absent in the media discourse was reference to human rights, including the right to food. A recent review of empirical articles on global health policy making showed the influence of moralization as a frame in shaping global health priorities (Shiffman and Shawar, 2022). The authors’ analysis showed how public and global health discourse on sexual and reproductive health have (quite successfully) employed a ‘moralization’ frame, depicting the issue as an ethical imperative using a human rights lens. This framing has been successful largely because it ensures an explicit emphasis on the obligations that state actors—as duty bearers—hold towards affected populations (Shiffman and Shawar, 2022). In the current review, while the findings also suggest an element of moralization in the treatment of food poverty and insecurity, the issue is rather depicted through the lens of charity, which of course precludes obligations for state actors and says nothing of the right to food. Re-framing discussions of food poverty and insecurity as a health and human rights issue may be a fruitful strategy for scholars, activists and others interested in addressing the problem, albeit such discursive shifts can take time. A salient example in this regard is the relatively recent change in discourse in respect of people with disabilities. The adoption of the Convention of the Rights of People with Disabilities (CRPD) by the UN in 2006 represented a definitive paradigm shift in how people with disabilities were viewed and treated by societies, moving from viewing them as objects of charity to people with rights which must be respected, protected and fulfilled. The right to food is set out in the International Covenant on Economic, Social and Cultural Rights, however a similar paradigm shift has yet to be widely realized (Ayala and Meier, 2017). Such a shift would potentially be expedited however if health promotors, researchers and other stakeholders interested in food poverty and insecurity moved towards adopting rights-based messaging when translating knowledge, including to policy-makers and the media.

For the public to receive a more realistic picture of food poverty and insecurity, the news media need to place greater attention on its multi-dimensional nature. This includes addressing food as a human right, acknowledging the social participatory aspects of food and eating and presenting the expressed views and experiences of those living in food poverty and insecurity. The positive framing of charitable food aid may convey the message to policy-makers that food poverty and insecurity are being effectively addressed through food charity, which may breed complacency among the general public about the urgency of the issue. Consequently, there is a need for more balanced media reporting that acknowledges the limitations of food charity and highlights the need for structural solutions to address this important public health issue. More critical news reporting will help foster an informed public who can demand government action to overcome the root causes of food poverty and insecurity. Another factor that could lead to more effective engagement with food poverty and insecurity is the fostering of constructive journalism approaches. Constructive journalism argues for socially responsible journalism that centres a public-oriented perspective, which often encompasses not just treating a news story through the lens of traditional ‘news values’ but offering pragmatic solutions to social issues (Gyldensted, 2015; Haagerup, 2015). In this sense, constructive journalism fosters an approach that journalism can help people and communities act on problems instead of just informing them (McIntyre and Gyldensted, 2018).

While there has been growing recognition of the importance of media framing of public health issues (Anderson *et al*., 2009; Gollust *et al*., 2019; Rowbotham *et al*., 2019, 2020; Kite *et al*., 2022), 10 studies over a 28-year period suggests that there is a paucity of research on news media framing of food poverty and insecurity. Furthermore, the review findings highlight much of the research is of poor methodological quality. Thus, there is a need for further studies of higher quality in this area. In particular, the review findings suggest the need for greater adherence to reporting guidelines for specific study designs to improve the completeness and transparency of this research. As newspapers were the only type of news media identified in this review, future research could consider the advent of online news media which has grown exponentially in the last number of years. As the traditional newspaper industry is transforming as a result of this ever-evolving digital media landscape, many newspaper publishing houses have started to produce news via online digital editions and websites. Additionally, ‘online only’ news sites and applications have also emerged, with their own journalistic routines and production practices. The volume of news media, wrought by digital media, presents new methodological challenges to consider when researching news framing. Methodological challenges aside, this research should also investigate framing of food poverty and insecurity in other news media platforms, such as broadcast and social media. The views of journalists and how they report and portray food poverty and insecurity is also an area for future research. Future research should also explore any differences in national and local news media coverage of food poverty and insecurity. In addition to media representation study, future research ought to explore both policy maker and public perspectives on the influences of news media coverage on food poverty and insecurity related actions at various levels.

### Limitations

Although the current review follows the recently developed Cochrane recommendations and minimum standards for conducting rapid reviews (Garritty *et al*., 2020), a number of limitations must be acknowledged. First, database searching of peer-reviewed articles was limited to three databases and included only English language studies. Furthermore, the search did not include unpublished and grey literature. These search restrictions may have introduced meta-bias, namely, selection, publication and language bias. Second, the risk of selection bias may have been introduced with two independent reviewers performing study selection and quality appraisal only on a sub-set of studies. Furthermore, a single reviewer conducted data extraction which may have introduced bias. Third, despite undertaking and reporting on quality assessment, all studies were synthesized equally in order to provide a literature summary. Therefore, evidence from studies may have been given undue weight and others underemphasized. Finally, limitations of the studies included in the review, including the predominant poor methodological quality, newspapers being the only type of news media examined and the limited number of high-income countries in which studies were conducted, may limit the internal and external validity of the review findings. All these limitations should be considered when interpreting the review findings.

## CONCLUSION

This review examined the evidence base for how food poverty and insecurity are represented in the news media. The review findings highlight a paucity of research on news media framing of food poverty and insecurity over the past three decades, with research solely focused on newspapers and mostly that of national print newspapers. The findings of the evidence synthesis revealed a largely absent nuanced understanding of food poverty and insecurity, with the problem often defined by food bank use and the consequences mainly focused on physical health. The causes of food poverty and insecurity were attributed less to individual failings than to the causes of poverty more generally (i.e. structural factors). Print media reinforced charitable solutions to food poverty and insecurity, with little critical analysis of such solutions. A significant presence of the deserving/undeserving stereotyping of food aid recipients was evident. Individuals’ experiences of food poverty and insecurity were, by and large, under-represented by the newspaper media. The findings of this review indicate that a major shift in print media discourse on food poverty and insecurity is required. It needs to move away from normalization and de-politicization of charitable food aid to the acknowledgement of food as a human right and the need for government accountability. Adopting constructive journalism techniques in news coverage may help with more effective engagement with food poverty and insecurity. Future research should investigate framing of food poverty and insecurity in online news media and other platforms, such as broadcast media.

## Supplementary Material

daad188_suppl_Supplementary_File_S1

daad188_suppl_Supplementary_File_S2

daad188_suppl_Supplementary_File_S3

daad188_suppl_Supplementary_File_S4
