# Supplementary material for: News media framing of food poverty and insecurity in high-income countries: a rapid review
Source: Health Promot Int. 2023 Dec 27;38(6):daad188. doi: 10.1093/heapro/daad188 (PMC10752350; doi:10.1093/heapro/daad188)
Supplement: daad188_suppl_Supplementary_File_S2 [file daad188_suppl_supplementary_file_s2.docx]

**Additional file 2.** Review search strategy

1. **Ovid Medline**

| 1  2  3  4  5  6  7  8  9  10  11  12  13  14  15  16  17  18  19  20  21  22 | food poverty.mp.  exp Food Insecurity/  food insecur*.mp.  food insufficien*.mp.  food deprivation.mp.  food bank*.mp.  food charit*.mp.  food assistance/  food assistance.mp.  nutrition assistance.mp  (access or availabilit*) adj4 food*  hunger  heat adj1 eat  1 or 2 or 3 or 4 or 5 or 6 or 7 or 8 or 9 or 10 or 11 or 12 or 13  exp communications media/  media.mp.  news*.mp.  magazine*.mp.  (television or tv).mp.  radio.mp.  15 or 16 or 17 or 18 or 19 or 20  14 and 21 |
| --- | --- |

1. **Scopus**

( TITLE-ABS-KEY ( radio )  OR  TITLE-ABS-KEY ( television  OR  tv )  OR  TITLE-ABS-KEY ( magazine* )  OR  TITLE-ABS-KEY ( news* )  OR  TITLE-ABS-KEY ( media ) )  AND  ( TITLE-ABS-KEY ( "food poverty" )  OR  TITLE-ABS-KEY ( "food insecur*" )  OR  TITLE-ABS-KEY ( "food insufficien*" )  OR  TITLE-ABS-KEY ( "food deprivation" )  OR  TITLE-ABS-KEY ( hunger )  OR  TITLE-ABS-KEY ( "food bank*" )  OR  TITLE-ABS-KEY ( "food charit*" )  OR  TITLE-ABS-KEY ( "Heat or Eat" )  OR  TITLE-ABS-KEY ( access*  W/4  food )  OR  TITLE-ABS-KEY ( "food assistance" )  OR  TITLE-ABS-KEY ( "nutrition assistance" ) )

1. **EBSCO APA PsycINFO**

| S1 | AB "food poverty" OR TI "food poverty" OR KW "food poverty" |
| --- | --- |
| S2 | AB "food insecur*" OR TI "food insecur*" OR KW "food insecur*" |
| S3 | MM "Food Insecurity" |
| S4 | AB "food insufficien*" OR TI "food insufficien*" OR KW "food insufficien*" |
| S5 | AB "food deprivation" OR TI "food deprivation" OR KW "food deprivation" |
| S6 | AB "food bank*" OR TI "food bank*" OR KW "food bank*" |
| S7 | AB "food charit*" OR TI "food charit*" OR KW "food charit*" |
| S8 | AB "food assistance" OR TI "food assistance" OR KW "food assistance" |
| S9 | AB "nutrition assistance" OR TI "nutrition assistance" OR KW "nutrition assistance" |
| S10 | AB ( (access* or availabilit*) N4 food* ) OR TI ( (access or availabilit*) N4 food* ) OR KW ( (access or availabilit*) N4 food* ) |
| S11 | AB heat N1 eat OR TI heat N1 eat OR KW heat N1 eat |
| S12 | AB hunger OR TI hunger OR KW hunger |
| S13 | S1 OR S2 OR S3 OR S4 OR S5 OR S6 OR S7 OR S8 OR S9 OR S10 OR S11 OR S12 |
| S14 | DE "Communications Media" OR DE "Audiovisual Communications Media" OR DE "Audiotapes" OR DE "Digital Video" OR DE "Educational Audiovisual Aids" OR DE "Films" OR DE "Photographs" OR DE "Radio" OR DE "Television" OR DE "Television Advertising" OR DE "Videotapes" OR DE "Digital Media" OR DE "Databases" OR DE "Digital Images" OR DE "Digital Information" OR DE "Digital Video" OR DE "Streaming Technology" OR DE "Mass Media" OR DE "Digital Media" OR DE "Films" OR DE "News Media" OR DE "Print Media" OR DE "Radio" OR DE "Television" OR DE "Multimedia" OR DE "Audiovisual Communications Media" OR DE "Social Media" OR DE "Online Social Networks" OR DE "Telecommunications Media" OR DE "Radio" OR DE "Telephone Systems" OR DE "Television" OR DE "Wireless Technologies" |
| S15 | TI media OR AB media OR KW media |
| S16 | TI news* OR AB news* OR KW news* |
| S17 | TI magazine* OR AB magazine* OR KW magazine* |
| S18 | TI ( (television or tv) ) OR AB ( (television or tv) ) OR KW ( (television or tv) ) |
| S19 | TI radio OR AB radio OR KW radio |
| S20 | S14 OR S15 OR S16 OR S17 or S18 or S19 |
| S21 | S13 AND S20 |
