# Supplementary material for: News media framing of food poverty and insecurity in high-income countries: a rapid review
Source: Health Promot Int. 2023 Dec 27;38(6):daad188. doi: 10.1093/heapro/daad188 (PMC10752350; doi:10.1093/heapro/daad188)
Supplement: daad188_suppl_Supplementary_File_S3 [file daad188_suppl_supplementary_file_s3.docx]

|  | **Screening Questions** | | **Qualitative Studies** | | | | |  |
| --- | --- | --- | --- | --- | --- | --- | --- | --- |
| First author, publication year | S1. Are there clear research questions? | S2. Do the collected data allow to address the research questions? | 1.1. Is the qualitative approach appropriate to answer the research question? | 1.2. Are the qualitative data collection methods adequate to address the research question? | 1.3. Are the findings adequately derived from the data? | 1.4. Is the interpretation of results sufficiently substantiated by data? | 1.5. Is there coherence between qualitative data sources, collection, analysis and interpretation? | Overall Quality Score |
| Yau *et al*., 2021 | Yes | Yes | Yes | Yes | Yes | Yes | Yes | 100% |
| Knight *et al*, 2018 | Yes | Yes | Yes | Yes | Yes | No | Yes | 80% |
| Marin-Murillo *et al*., 2020 | Yes | No | Yes | No | Can't tell | Yes | Can’t tell | 40% |
| Price *et al*., 2020 | Yes | Yes | No | Yes | No | Yes | No | 40% |
| Wells and Caraher, 2014 | Yes | Yes | No | Yes | Can’t tell | Yes | Can’t tell | 40% |
| Smith-Carrier, 2021 | Yes | Can’t tell | Yes | Can't tell | Yes | Yes | Yes | 80% |
| Tikka, 2019 | Yes | No | Yes | No | Can’t tell | Can’t tell | No | 20% |
| Mejia *et al*., 2022 | Yes | Yes | Can't tell | Yes | Can't tell | No | Can’t tell | 20% |
| Henderson and Foley, 2010 | Yes | Can’t tell | Yes | Can't tell | Can't tell | Yes | Can’t tell | 40% |

**Additional file 3.** Methodological quality criteria scoring using the Mixed-Methods Appraisal Tool (MMAT) version 2018

|  | **Screening Questions** | | **Quantitative Descriptive Studies** | | | | |  |
| --- | --- | --- | --- | --- | --- | --- | --- | --- |
| First author, publication year | S1. Are there clear research questions? | S2. Do the collected data allow to address the research questions? | 4.1. Is the sampling strategy relevant to address the research question? | 4.2. Is the sample representative of the target population? | 4.3. Are the measurements appropriate? | 4.4. Is the risk of nonresponse bias low? | 4.5. Is the statistical analysis appropriate to answer the research question? | Overall Quality Score |
| Collins *et al*., 2021 | Yes | Yes | Yes | No | Yes | Yes | No | 60% |

**References**

Collins, P. A., Gaucher, M., Power, E. M., and Little, M. H. (2016) Implicating municipalities in addressing household food insecurity in Canada: A pan-Canadian analysis of news print media coverage. *Canadian Journal of Public Health. Revue Canadienne de Sante Publique,* **107**, e68-e74. doi: [10.17269/cjph.107.5231](https://dx.doi.org/10.17269/cjph.107.5231).

Henderson, J., and Foley, W. (2010) Brace yourselves: Reporting of rising food costs in the Australian print media. *Australian Journal of Social Issues,* **45**, 477-492. doi: 10.1002/j.1839-4655.2010.tb00193.x.

Knight, A., Brannen, J., O'Connell, R., and Hamilton, L. (2018) How do children and their families experience food poverty according to UK newspaper media 2006-15? *Journal of Poverty and Social Justice,* **26**, 207-223. doi: 10.1332/175982718X15200701225223.

Marín-Murillo, F., Armentia-Vizuete, J. I., Marauri-Castillo, I., and Rodríguez-González, M. M. (2020) Food accessibility on digital press: Framing and representation of hunger in Spain. *Revista Latina de Comunicacion Social,* **75**, 169-187. doi: 10.4185/RLCS-2020-1421.

Mejia, P., Mahmood, H., Perez-Sanz, S. B., Garcia, K., and Dorfman, L. A. (2022) "People Like Us": News Coverage of Food Assistance During the COVID-19 Pandemic. *Health Equity,* **6**, 367-374. doi: [10.1089/heq.2022.0001](https://dx.doi.org/10.1089/heq.2022.0001).

Price, C., Barons, M., Garthwaite, K., and Jolly, A. (2020) 'The do-gooders and scroungers': Examining narratives of foodbank use in online local press coverage in the West Midlands, UK. *Journal of Poverty and Social Justice,* **28**, 279-298. doi: 10.1332/175982720X15905998323834.

Smith-Carrier, T. (2021) ‘The (charitable) pantry is bare’: a critical discourse analysis of Christmas food hamper programs in Canada. *Critical Policy Studies,* **15**, 90-106. doi: 10.1080/19460171.2020.1722190.

Tikka, V. (2019) Charitable food aid in Finland: from a social issue to an environmental solution. *Agriculture and Human Values,* **36**, 341-352. doi: 10.1007/s10460-019-09916-3.

Wells, R., and Caraher, M. (2014) UK print media coverage of the food bank phenomenon: From food welfare to food charity? *British Food Journal,* **116**, 1426-1445. doi: 10.1108/BFJ-03-2014-0123

Yau, A., Singh-Lalli, H., Forde, H., Keeble, M., White, M., and Adams, J. (2021) Newspaper coverage of food insecurity in UK, 2016–2019: a multi-method analysis. *BMC Public Health,* **21**, 1201. doi: 10.1186/s12889-021-11214-9.
