# Supplementary material for: News media framing of food poverty and insecurity in high-income countries: a rapid review
Source: Health Promot Int. 2023 Dec 27;38(6):daad188. doi: 10.1093/heapro/daad188 (PMC10752350; doi:10.1093/heapro/daad188)
Supplement: daad188_suppl_Supplementary_File_S4 [file daad188_suppl_supplementary_file_s4.docx]

| **Additional file 4.** News media framing of food poverty and insecurity mapped to Entman’s framing theory | | | | | |
| --- | --- | --- | --- | --- | --- |
| **Studies** | **Definition of Problem** | **Causal Attribution** | **Moral Evaluation** | **Treatment Recommendation** | **Actors/Voices** |
| Yau *et al*., 2021 | Food insecurity largely defined as insufficient food quantity and by its symptoms, such as hunger and meal skipping.  Some reference to diet quality and social participation.  Food bank use as synonymous with food insecurity  ‘Holiday hunger’ commonly used to define the extent and timing of child food insecurity  Food insecurity (and food bank use) used to illustrate extreme poverty and destitution  A range of consequences for individuals:   - Mental, physical and social health - For children - educational consequences alongside a lack of future opportunities - For families - adults cut back to ensure children do not have to | Insufficient income frequently cited (due to low wages and unstable incomes among the 'working poor' and high cost of living)  Upstream drivers were governmental (regressive policies - austerity and welfare reform)  Some reference to individual responsibility (such as money management) | Food insecurity incompatible with modern day living - reference to periods of poverty in British history  Reference to ‘working poor’ at risk  Public outrage  Redistribution of 'food waste' initiatives presented as a double win for both environment and food insecurity  Some articles critique food banks - not sustainable and address symptoms rather root cause of food insecurity  Government reluctance to take action to tackle food insecurity was portrayed across articles - denial of problem and their responsibility | Existing solutions predominately focused on charitable food aid - food banks and redistribution of food waste  Some articles note food banks providing budgeting tips and recipes (skills-based) alongside food aid  Calls for government action - examples of proposed structural solutions include welfare reform (e.g., increase in benefit payment, extend eligibility for welfare support), improved worker rights (e.g., increase minimum wage), affordable childcare | General public  Advocacy groups  Charities  Politicians  Individuals experiencing food insecurity |
| Knight *et al*., 2018 | Food banks are a metonym for food poverty  Food bank use linked with malnutrition due to poor food quality | Food bank rise largely attributed to economic difficulties relating to recession, income stagnation, welfare reform and increasing food prices.  As UK started to move out of recession - debate shifted to whether food bank rise due to benefit sanctions or whether the supply of food banks was fuelling demand  Some reference to individual responsibility (such as lack of cooking skills) | Question over statistics on food poverty  - government accuses the Trussell Trust of ‘scaremongering’  Sense of shock – public outrage  Debate over food bank recipient deservingness - those that deserve it (deemed fit for work and 'couldn't make ends meet') versus those not deserving ('conman', casting doubt on the needs and motives of food bank users)  Food banks as a driver ('enemy') and then solution (potential 'partner' of welfare state)  Food banks a potential 'partner' of welfare state and an example of the ‘Big Society’  Food banks creating 'welfare dependency'  Children’s' right to food | Food banks as a solution | Charities (namely the Trussell Trust)  Journalists  Government ministers  Anti-poverty campaigners (working from personal experience)  Church leaders  Other public figures  Voice of parents and children living in food poverty is largely absent |
| Price *et al*., 2020 | Food bank use linked with poverty  Emotional consequences - feelings of desperation, shame, stigma and embarrassment experienced by food bank users | Food bank use linked with issues with the welfare system (such as inadequate social benefit, benefit sanctions) resulting in insufficient income | Food bank recipients deserving of assistance – empathy and compassion in media reporting  Food bank use linked with the 'poor' requiring assistance  Narratives surrounding the ‘Big Society’ and citizens playing an active role in their community (i.e., donating to food banks)  Positive view of food banks - minimal critical analysis of food bank models | Food banks as a solution | Food bank users |
| Wells and Caraher, 2014 | Food bank use linked with poverty | Unemployment and high cost of living (including food prices)  Debate over whether the changes in welfare provision or the proliferation of food banks is the reason for the increase in food bank use  Some reference to individual responsibility (such as money management) | The existence of food banks incompatible with modern day living (referred to as shocking) - reference to periods of poverty in British history  Food banks a new term - portrayed as something exotic and unknown in earlier reporting (before 2013)  Reporting over the Christmas period driven by a sense of Christian care and appeals for compassion and/or donations  Positive view of food banks - minimal critical analysis of food bank models  Debate over food bank recipient deservingness (generated by politicians) | Food banks (including those which redistribute food waste) as a solution – the Trussell Trust as the model of operation | The Trussell Trust - a key source and gatekeeper of information on food banks  Volunteers - proxy voice for food bank users  Politicians (in and out of government)  Church leaders (particularly the Church of England)  Celebrities (including a former food bank user)  Activists  Limited quotes from food bank users. |
| Collins, *et al*., 2016 | No data | No data | Tone more negative when implicating senior governments (federal, provincial/territorial) in actions to address food insecurity and more neutral and positive when local/municipal governments were profiled.  Positive framing of local-level, food-based programs (e.g., community gardens, community kitchens, good food boxes) | Frequently profiled local-level (i.e., neighbourhood- or municipal-level) programs and implicated municipal government to take action to address food insecurity  Community gardens/urban agriculture was the most profiled food-based program at local level, followed by food banks/meal programs | No data |
| Smith-Carrier, 2021 | No data | Poverty as derived from a lack of education (specifically, budgeting knowledge)  No reference to structural causes of increasing food charity use | Food charity recipients cast as 'desperate' and 'needy' - responsible for their poor circumstances  Parents of young children, particularly lone mothers and university students are deemed more deserving of assistance due to their care of children or being considered future employees/workers  Expectations of gratitude and preferred behaviours (e.g., express their indigence, demonstrate the ‘Christmas spirit’ expected of them) from food charity recipients  Assumed adequacy and venerated practice of food charity in the wake of increasing need  Volunteers (givers/donors) being framed as the saviour or rescuer of 'poor' people, while the moral stature of recipients appears suspect  Rewards for volunteers (feelings of joy, pleasure and fulfilment) evident in reporting, while the emotional costs for recipients omitted | Charitable assistance is the solution; however, missing discourse around the adequacy of charitable solution  Little dialogue surrounded the need for government action to redress structural inequalities | Do not identify voices/actors; however, quotes from food bank representatives presented in the results |
| Mejia *et al*., 2022 | Limited connection between food access and health | COVID-19 pandemic as causing hunger as a result of people losing jobs and struggling to pay bills  Few articles link structural inequities to food insecurity | Before the pandemic, food assistance programs described as vulnerable to misuse by those undeserving – images include people of colour  During the pandemic, recipients of food assistance (many referenced families) were portrayed empathetically as “people like us” - images include unrelated groups of adults, both white and people of colour  Recipients of food assistance during the pandemic are presented as honest people that are seeking work  Challenges faced by charitable initiatives (e.g., funding)  Difficulties in accessing charitable food aid (e.g., complex application process) | Food assistance programs  Support for government policy solutions more than tripled during the pandemic | Government representatives (local, state and federal)  Representatives of food assistance organisations  Anti-hunger advocates |
| Henderson and Foley, 2010 | Food insecurity (via increasing food prices) represents a threat to groups who are economically vulnerable  Health and nutritional consequences associated with food insecurity (via increasing food prices) | Rising food prices (and overall cost of living) | The ‘average Australian’ impacted by rising food prices with the villain primarily presented as major supermarkets; however, supermarkets are subsequently de-villainized following an inquiry into food prices which finds prices outside the control of retailers | Political solutions proposed (GROCERYchoice website, a site that allowed comparison of prices across supermarkets and the introduction of unit pricing) to increase personal responsibility  Cost-saving strategies proposed – home brands, buying in bulk, packing lunch etc. | Media - a claim maker in relation to the threat posed by rising food costs  Government officials/politicians |
| Tikka, 2019 | Charitable food aid as an emblem of poverty | Increase use of charitable food aid linked with insufficient income (due to austerity measures during economic recession); however, when charitable food aid is (re)framed as a circular economy model, the root causes for food insecurity are not addressed | Reporting about charitable food aid was mainly neutral  However, when charitable food aid (re)framed as a circular economy model which solves the issue of excess in the food supply chain - perception of charitable food aid is mainly favourable  Discussions shift from poverty problem (social problem) to reducing food waste (environmental problem)  Charitable food aid only gained policy relevance when linked with food waste | Redistribution of surplus food from the retail and food industry to food charities - to resolve both food insecurity and food waste | Media – key voice  Church leaders  Politicians  Private sector  Poor representation of food charity organisations (leaders/volunteers) and non-governmental organisations  Recipients of food aid not quoted |
| Marín-Murillo *et al*., 2020 | Hunger largely defined by poverty  Solidarity initiatives (such as food banks) take on the meaning of hunger  Consequences of hunger include malnutrition, obesity and shorter life expectancy due to poor diet quality | Hunger/food accessibility not generally viewed as a structural problem but a problem of individual nature | Recipients of food assistance presented as mainly families that are “vulnerable”, "disadvantaged", "in need" or simply "poor"  Recipients presented as passive subjects of circumstances that seem arbitrary  Interest driven collaborations are presented, where the private sector contributes to the cause at the same time as advertising themselves – ‘branded aid’  Challenges faced by solidarity initiatives (e.g., getting enough volunteers) and their successes (e.g., awards they have won)  Right to food only referenced in one article | Articles which look at political solutions are a minority  Solidarity initiatives (coming from non-governmental organisations, private companies and individuals dedicating their efforts to supply food) has replaced institutional responsibility | Non-governmental organisations  Private sector – banks, supermarkets, food product companies  Voice of those affected by hunger and using solidarity initiatives largely absent |

**References**

Collins, P. A., Gaucher, M., Power, E. M., and Little, M. H. (2016) Implicating municipalities in addressing household food insecurity in Canada: A pan-Canadian analysis of news print media coverage. *Canadian Journal of Public Health. Revue Canadienne de Sante Publique,* **107**, e68-e74. doi: [10.17269/cjph.107.5231](https://dx.doi.org/10.17269/cjph.107.5231).

Henderson, J., and Foley, W. (2010) Brace yourselves: Reporting of rising food costs in the Australian print media. *Australian Journal of Social Issues,* **45**, 477-492. doi: 10.1002/j.1839-4655.2010.tb00193.x.

Knight, A., Brannen, J., O'Connell, R., and Hamilton, L. (2018) How do children and their families experience food poverty according to UK newspaper media 2006-15? *Journal of Poverty and Social Justice,* **26**, 207-223. doi: 10.1332/175982718X15200701225223.

Marín-Murillo, F., Armentia-Vizuete, J. I., Marauri-Castillo, I., & Rodríguez-González, M. M. (2020) Food accessibility on digital press: Framing and representation of hunger in Spain. *Revista Latina de Comunicacion Social,* **75**, 169-187. doi: 10.4185/RLCS-2020-1421.

Mejia, P., Mahmood, H., Perez-Sanz, S. B., Garcia, K., & Dorfman, L. A. (2022) "People Like Us": News Coverage of Food Assistance During the COVID-19 Pandemic. *Health Equity,* **6**, 367-374. doi: [10.1089/heq.2022.0001](https://dx.doi.org/10.1089/heq.2022.0001).

Price, C., Barons, M., Garthwaite, K., & Jolly, A. (2020) 'The do-gooders and scroungers': Examining narratives of foodbank use in online local press coverage in the West Midlands, UK. *Journal of Poverty and Social Justice,* **28**, 279-298. doi: 10.1332/175982720X15905998323834

Smith-Carrier, T. (2021) ‘The (charitable) pantry is bare’: a critical discourse analysis of Christmas food hamper programs in Canada. *Critical Policy Studies,* **15**, 90-106. doi: 10.1080/19460171.2020.1722190

Tikka, V. (2019) Charitable food aid in Finland: from a social issue to an environmental solution. *Agriculture and Human Values,* **36**, 341-352. doi: 10.1007/s10460-019-09916-3

Wells, R., and Caraher, M. (2014) UK print media coverage of the food bank phenomenon: From food welfare to food charity? *British Food Journal,* **116**, 1426-1445. doi: 10.1108/BFJ-03-2014-0123

Yau, A., Singh-Lalli, H., Forde, H., Keeble, M., White, M., and Adams, J. (2021) Newspaper coverage of food insecurity in UK, 2016-2019: a multi-method analysis. *BMC Public Health,* **21**, 1201. doi: [10.1186/s12889-021-11214-9](https://dx.doi.org/10.1186/s12889-021-11214-9)
